# Omega-3 Fatty Acids Supplementation Improve Nutritional Status and Inflammatory Response in Patients With Lung Cancer: A Randomized Clinical Trial

**DOI:** 10.3389/fnut.2021.686752

**Published:** 2021-07-30

**Authors:** Mingjin Cheng, Shengqiang Zhang, Chengdong Ning, Qianlun Huo

**Affiliations:** ^1^Department of Cardiothoracic Surgery, The Lu'an Hospital Affiliated to Anhui Medical University, Lu'an, China; ^2^Department of Cardiothoracic Surgery, The Lu'an People's Hospital, Lu'an, China

**Keywords:** omega-3 fatty acids, lung cancer, nutrition, inflammation, supplement

## Abstract

**Background and Aims:** Clinical studies have reported positive results with omega-3 supplements in patients with cancer. This study aimed to evaluate the efficacy of omega-3 fatty acid supplementation in improving the nutritional status and inflammatory markers of patients with lung cancer.

**Methods:** In a randomized, double-blind, parallel design trial, 60 patients with lung cancer at nutritional status/risk based on the Nutrition Risk Screening 2002 were randomized to be allocated to two study groups, receiving omega-3 fatty acid supplements [eicosapentaenoic acid (EPA) 1.6 g and docosahexaenoic acid (DHA) 0.8 g] or placebo for 12 weeks. Anthropometric measurements [weight, body mass index (BMI), the circumference of the upper arm, and skinfold thickness of triceps], nutrition-based laboratory indices (hemoglobin, albumin, triglyceride, and cholesterol), and inflammatory markers [C-reactive protein (CRP), tumor necrosis factor alpha (TNF-α), and interleukin 6 (IL-6)] were measured before and after the intervention as study outcomes.

**Results:** No significant difference between the two study groups was observed regarding basic characteristics and study outcomes. Compared with placebo group, omega-3 fatty acid supplementation group showed significant higher weight (66.71 ± 9.17 vs. 61.33 ± 8.03, *p* = 0.021), albumin (4.74 ± 0.80 vs. 4.21 ± 0.77, *p* = 0.013), and triglyceride (130.90 ± 25.17 vs. 119.07 ± 14.44, *p* = 0.032). Inflammatory markers were significantly reduced in omega-3 group compared to placebo (CRP 1.42 ± 0.63 vs. 3.00 ± 1.05, *p* = 0.001 and TNF-α 1.92 ± 0.65 vs. 4.24 ± 1.19, *p* = 0.001). No significant difference was observed between the two study groups regarding changes in BMI, the circumference of the upper arm, skinfold thickness of triceps, triglyceride, cholesterol, and IL-6 (*p* > 0.05).

**Conclusions:** Omega-3 fatty acid supplementation can improve nutritional status and suppress the systemic inflammatory response in patients with lung cancer.

**Clinical Trial Registration:**www.socialscienceregistry.org, identifier: AEARCTR-0007165.

## Introduction

Lung cancer is by far the leading cause of death from cancer among both men and women, making up almost 25% of all deaths from cancer (1, 2). Lung cancer is reported to account for more than 131,000 annual mortality rates in the United States, and the rate is estimated to be 1.8 million worldwide (3). Mostly associated with the rate of smoking, the highest incidence rates of lung cancer among men are in Europe, particularly in Eastern European countries and Western Asia (3). In most parts of the world, tobacco use is the main cause of lung cancer, followed by other risk factors such as air pollution, radon, and several occupational agents (4, 5).

Taking into consideration the lack of definite curative treatment in most patients with lung cancer, many investigations are focused on palliative care and improvement of quality of life in these patients (6–8). The nutritional status of these patients is among the important factors affecting their quality of life (9, 10). Moreover, evidence suggests that impaired nutritional status in patients with lung cancer negatively affects the immune function of patients leading to a higher rate of infection and complications that end in increased mortality (11, 12). Therefore, investigations exploring the potential options for improving the nutritional status of these patients are the focus of research studies in the field of lung cancer (13–15).

There is a growing body of evidence that omega-3 fatty acid supplementation improves nutritional status in patients with cancer through different mechanisms (16, 17). Omega-3 fatty acids are polyunsaturated fatty acids (PUFAs) characterized by the presence of a double bond three atoms away from the terminal methyl group in their chemical structure (18). They are widely found in natural oils of edible seeds and marine products and play important role in lipid metabolism in humans (19, 20). Omega-3 fatty acids are shown to suppress systemic inflammatory (21) and oxidative responses (22), improve the appetite of patients, and enhance weight gain in cachectic patients with cancer (23, 24). Omega-3 fatty acids are evaluated for their beneficial effects in different types of cancer including colorectal cancer, oral squamous cell cancer, breast cancer, and hematologic cancer with promising results (23). Besides the mentioned evidence, there is not yet enough evidence to definitely support the use of omega-3 fatty acid supplementation in these patients and more randomized, controlled clinical studies considering relevant outcomes, adequate sample size, and proper doses are suggested (25).

With regard to the suggested role of omega-3 fatty acids in improving the nutritional status of patients with cancer through anti-inflammatory mechanisms, this study aimed to evaluate this effect in patients with lung cancer in a randomized controlled trial.

## Methods

### Trial Design

The study was designed as a double-blinded placebo-controlled randomized clinical trial including two arms with parallel design. No change was made in methods after the commencement of the trial.

### Participants

Sixty patients with the pathology-confirmed diagnosis of lung cancer attending the oncology clinic from May 2019 to September 2020, with a Nutrition Risk Screening 2002 score of equal or more than 4 (at risk or higher), were evaluated for inclusion in the study. Patients with allergy to fish or fish oil, diabetes mellitus uncontrolled chronic cardiac and renal disease, gastrointestinal diseases, inability to take oral feeding, and on other nutritional programs or supplements were excluded from the study.

### Sample Size

The sample size was calculated considering a one-sided significance level of 0.05 and a power of 0.80 as for the investigational design based on results of previous studies. Finally, 60 patients were equally assigned to the omega-3 fatty acid supplementation and placebo groups.

### Interventions

Omega-3 fatty acid supplementation gel capsules [eicosapentaenoic acid (EPA) 1.6 g/day and docosahexaenoic acid (DHA) 0.8 g/day, derived from fish oil in gel capsules] or placebo gel capsules (sunflower oil) with a similar appearance (supplied by Sinadaro, China) were provided to the patients for 12 weeks of intervention. The dose was selected according to the previous study (26). Compliance of the patients was evaluated by a form marked on each day of taking capsules by the patient him/herself. The forms were checked in each visit by the investigator. Patients with more than three lost doses per month were excluded from the analysis.

All patients received nutrition counseling for the Nutrition Risk Screening and the measurement of nutritional values of the intake of food and beverage. Three-day dietary records were used to calculate the intake of food and beverage at baseline and at end of the intervention. Each 3-day record consisted of 2 weekdays and 1 weekend. Foods were measured by applying standard measuring cups and spoons. Patients were instructed in the nutrition counseling visit on the method of filling the form and asked to complete 1-day food intake boxes in the form at the same visit to confirm the understanding of the patient about the correct method of the form filling. A telephone number was given to all patients to ask their questions on filling the form. The prospective filled forms were gathered in the follow-up visit, and the registered data were analyzed by the nutritionist to calculate the nutritional values using a pre-defined table.

All the patients followed the standard protocol of their treatments for cancer. They were recommended to maintain routine physical activity and diet. The patients were instructed to report any observed adverse event to the researcher by telephone call.

### Outcomes

Anthropometric measurements [weight, body mass index (BMI), the circumference of the upper arm, and skinfold thickness of triceps], nutrition-based laboratory indices (hemoglobin, albumin, triglyceride, and cholesterol), and inflammatory markers [C-reactive protein (CRP), tumor necrosis factor alpha (TNF-α), and interleukin 6 (IL-6)] were measured before and after the intervention as study outcomes.

Anthropometric measurements that included body weight, the circumference of the upper arm, and skinfold thickness of triceps were performed as per the International Standards for Anthropometric Assessment Manual (27). Weight was measured when patients were fasting without shoes wearing the light same clothing for all measurements. The mid-arm of the right hand was used for the measurement of the circumference of the arm. Skinfold thickness of triceps was measured by applying Harpenden Caliper.

The CRP was measured using Human High Sensitivity C-Reactive Protein ELISA Kit (Anisan™, Tehran, Iran) and serum albumin using Albumin Kit (Novin Bio Kit™, Tehran, Iran). Serum TNF-α and IL-6 levels were measured by enzyme-linked immunosorbent assay (ELISA) kits, (Biosource Europe, Belgium) specified for human cytokines.

### Randomization, Blinding, and Allocation Concealment

Sixty eligible patients were randomly allocated to the two study groups by the clinic secretary based on a randomization list. The list was created by the block randomization method. The oncologist, nutritionist, and secretary all kept blind to the group assignments of patients. Patients also remained blind to the treatment groups by similarity in the shape, color, and size of gel capsules and bottles of omega-3 fatty acids and placebo.

### Statistical Analysis

Statistical analysis was undertaken using SPSS v.18.0 (IBM Corp., Armonk, NY, USA). The descriptive data were presented by means/SEM for quantitative data and numbers/percentage for qualitative data. Student's *t*-test and chi-square-test were used for statistical comparison of primary characteristics and outcomes within and between the drug and placebo groups. A *p-*value of <0.05 was considered significant.

### Ethical Considerations

The study protocol was approved by the Local Medical Ethics Committee of Anhui Medical University (Ethical Committee Code: EC/2156-763) to comply with the Declaration of Helsinki. The pre-determined method of the study was registered in the clinical trial registry (AEARCTR-0007165) https://www.socialscienceregistry.org/trials/7165.

## Results

### Study Flow

**Figure 1:**
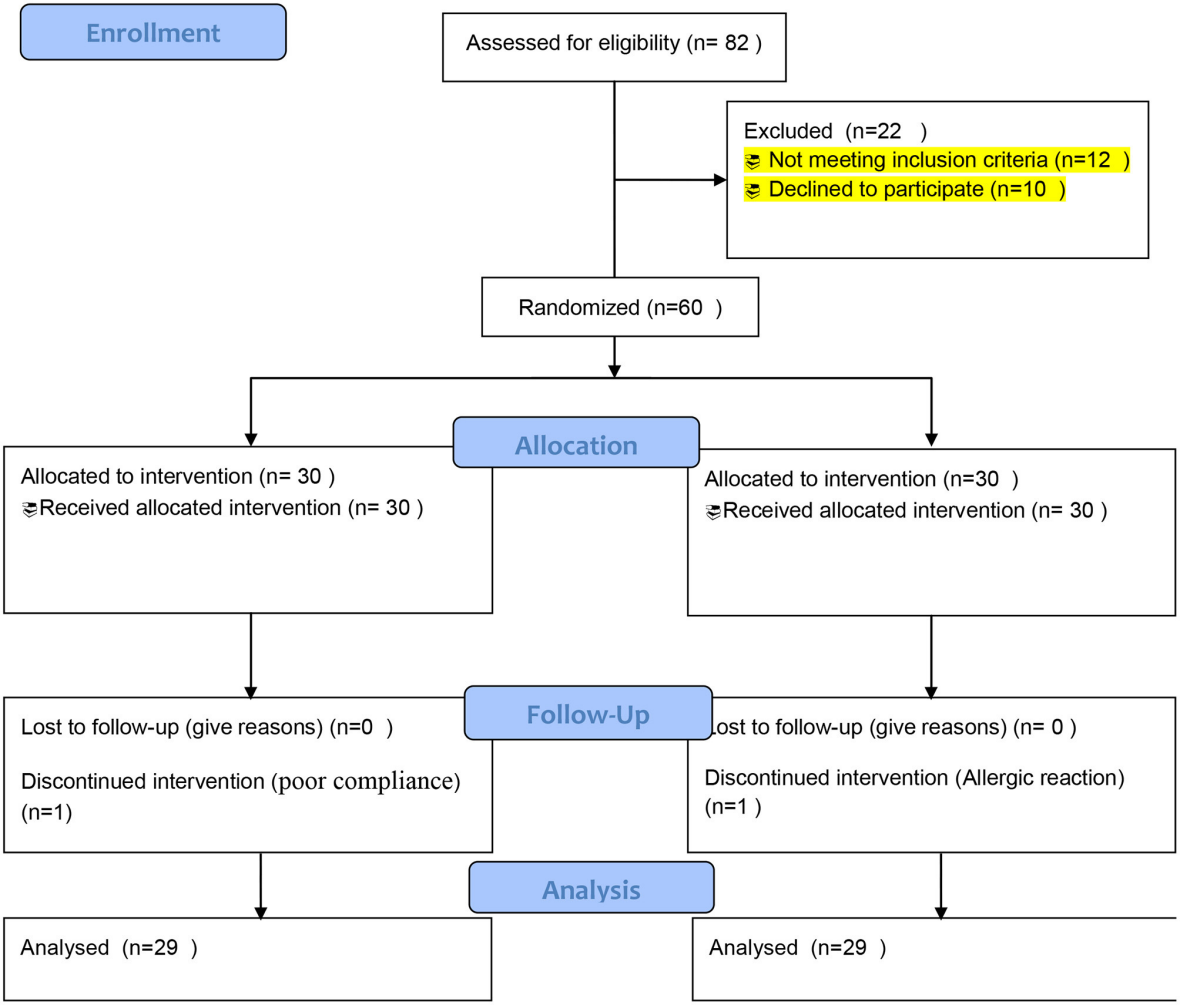
Consolidated standards of reporting trials (CONSORT) flow diagram of the enrollment and follow-up of the patients.

Eighty-two patients diagnosed with lung cancer at nutritional status/risk based on the Nutrition Risk Screening 2002 were evaluated for eligibility to participate in the study. A total of 60 eligible patients were randomized into omega-3 fatty acid supplementation and placebo groups from April 2019 to October 2020. One patient in the omega-3 group was excluded due to poor compliance with the drug in the study. Another patient in the placebo group was excluded from the study due to a self-report of some allergic reaction. Finally, 58 patients underwent final analysis. The study flow is summarized in Figure 1 as consolidated standards of reporting trials (CONSORT)-oriented flow diagram.

### Basic Characteristics

The enrolled patients in the study had no significant difference in baseline demographic and clinical characteristics including age, gender, Nutrition Risk Screening score, type of cancer, and the received chemotherapeutic treatment. The details of baseline characteristics of the patients in the trial are summarized in Table 1.

**Table 1:** Baseline demographic and clinical characteristics of patients with lung cancer in omega-3 fatty acid supplementation and placebo groups.

**Characteristics**	**Omega-3 (Mean ± SD) *n* = 29**	**Placebo (Mean ± SD) *n* = 29**	*P*-value^**^
Gender (female/male)	16/13	10/19	0.113
Age (year)	63.03 ± 5.32	64.55 ± 8.08	0.403
NRS^*^ score	4.31 ± 0.60	4.41 ± 0.63	0.525
**Type of lung cancer**			
Small cell lung cacner	2	3	0.726
Adenocarcinomas	13	12	
Squamous cell carcinomas	10	7	
Large cell carcinomas	2	5	
Others	2	2	
**Stage of lung cancer**			
Stage I NCSLC	2	3	
Stage II NCSLC	7	8	
Stage III NCSLC	12	10	
Stage IV NCSLC	6	5	
Stage limited SCLC	1	1	
Stage extensive SCLC	1	2	
**Small cell lung cancer**			
**Type of treatment**			
Cisplatin	14	15	
Cisplatin and docetaxel	13	11	0.682
Etoposide	2	3	

* *Nutrition Risk Screening*.

** *Chi-square-test for categorical variables, and t-test for quantitative variables*.

### Anthropometric Outcomes

There was a significant increase in the weight of the omega-3 group, which was observed through the trial (61.86 ± 10.02 vs. 66.71 ± 9.17, *p* = 0.049) without any significant change in their calorie intake (2,067 ± 338 vs. 2,133 ± 420, *p* = 0.512). The final weight of patients in the active intervention group was also significantly higher than the placebo group (66.71 ± 9.17 vs. 61.33 ± 8.03, *p* = 0.021). No other significant difference was observed between the two study groups regarding their BMI, the circumference of the arm, and skinfold thickness of triceps (*p* > 0.05; Table 2).

**Table 2:** Anthropometric outcomes of patients with lung cancer in omega-3 fatty acid supplementation and placebo groups.

**Characteristics**		**Omega-3** **(Mean ± SD)** ***n* = 29**	**Placebo** **(Mean ± SD)** ***n* = 29**	*P*-value^*^
Weight (Kg)	Before	61.86 ± 10.02	62.46 ± 8.88	0.808
	After	66.71 ± 9.17	61.33 ± 8.03	0.021
BMI (Kg/m^2^)	Before	20.04 ± 4.57	21.13 ± 4.48	0.363
	After	21.64 ± 4.59	20.81 ± 4.54	0.831
Upper arm circumference	Before	24.70 ± 2.91	24.70 ± 2.88	0.990
	After	25.86 ± 2.04	24.87 ± 2.68	0.120
Skinfold thickness	Before	12.43 ± 1.62	12.46 ± 1.60	0.948
	After	13.54 ± 1.81	12.71 ± 1.52	0.064
Calorie intake (Kcal/d)	Before	2,067 ± 338	2,061 ± 369	0.950
	After	2,133 ± 420	2,090 ± 434	0.703

* *indicates p-values for between groups comparisons*.

### Laboratory Indices of Nutritional Status

A significant increase in serum albumin (4.18 ± 1.06 vs. 4.74 ± 0.80, *p* = 0.027) and triglyceride (114.45 ± 20.74 vs. 130.90 ± 25.17, *p* = 0.008) was observed in the omega-3 group. The final values for albumin (4.74 ± 0.80 vs. 4.21 ± 0.77, *p* = 0.013) and triglyceride (130.90 ± 25.17 vs. 119.07 ± 14.44, *p* = 0.032) were also higher in the omega-3 group compared to the placebo group. Although the hemoglobin level was increased in the omega-3 supplementation period (9.92 ± 1.78 vs. 10.25 ± 1.45, *p* = 0.442), the change did not reach a level of statistical significance. The serum cholesterol level showed also no significant change in the study groups (Table 3).

**Table 3:** Laboratory indices of nutritional status with lung cancer in omega-3 fatty acid supplementation and placebo groups.

**Characteristics**		**Omega-3** **(Mean ± SD)** ***n* = 29**	**Placebo** **(Mean ± SD)** ***n* = 29**	*P*-value^*^
Hemoglobin (g/dL)	Before	9.92 ± 1.78	9.53 ± 2.27	0.469
	After	10.25 ± 1.45	9.60 ± 1.97	0.160
Albumin (g/dL)	Before	4.18 ± 1.06	4.31 ± 1.09	0.644
	After	4.74 ± 0.80	4.21 ± 0.77	0.013
Triglyceride (mg/d)	Before	114.45 ± 20.74	118.86 ± 22.04	0.436
	After	130.90 ± 25.17	119.07 ± 14.44	0.032
Cholesterol (mg/d)	Before	154.52 ± 48.46	150.62 ± 57.38	0.781
	After	153.28 ± 45.57	152.97 ± 45.19	0.979

* *indicates p-values for between groups comparisons*.

### Inflammatory Markers

A significant decrease in serum CRP (2.69 ± 1.34 vs. 1.42 ± 0.63, *p* = 0.001) and TNF-α (3.74 ± 1.69 vs. 1.92 ± 0.65, *p* = 0.001) was observed in the omega-3 group. The final values for CRP (1.42 ± 0.63 vs. 3.00 ± 1.05, *p* = 0.001) and TNF-α (1.92 ± 0.65 vs. 4.24 ± 1.19, *p* = 0.001) were also lower in omega-3 group compared to placebo group. Although the hemoglobin level was decreased in the omega-3 supplementation period (4.27 ± 0.88 vs. 4.15 ± 1.46, *p* = 0.706), the change did not reach a level of statistical significance. The serum cholesterol level showed also no significant change in the study groups (Table 4).

**Table 4:** Inflammatory markers of patients with lung cancer in omega-3 fatty acid supplementation and placebo groups.

**Characteristics**		**Omega-3** **(Mean ± SD)** ***n* = 29**	**Placebo** **(Mean ± SD)** ***n* = 29**	*P*-value^*^
CRP^*^ (mg/L)	Before	2.69 ± 1.34	2.79 ± 1.24	0.769
	After	1.42 ± 0.63	3.00 ± 1.05	0.001
TNFα^**^(pg/mL)	Before	3.74 ± 1.69	3.87 ± 1.78	0.775
	After	1.92 ± 0.65	4.24 ± 1.19	0.001
IL-6^***^(pg/mL)	Before	4.27 ± 0.88	4.23 ± 0.93	0.852
	After	4.15 ± 1.46	4.20 ± 1.48	0.901

* *C-reactive protein*.

** *Tumor necrosis factor alpha*.

*** *Interleukin 6*.

## Discussion

This study provided preliminary evidence for the beneficial effects of omega-3 fatty acid supplementation in the nutritional status of patients with lung cancer. Among anthropometric and nutrition-based laboratory indices, weight and serum albumin levels showed to be improved in our investigation. The observed decrease in inflammatory markers of the patients besides the previous evidence on the role of systemic inflammation in the nutritional status of patients with cancer supports the anti-inflammatory effect of omega-3 fatty acids as an important mediator of the observed clinical effect in improving the nutritional status of the patients.

The results of our study complied with other clinical trials on the effect of omega-3 fatty acids on the nutritional status of patients with different types of cancer. Nemati et al. have reported the positive effect of omega-3 fatty acid supplementation on nutritional status in patients with gastric cancer undergoing chemotherapy. They have assigned 30 patients to receive 3 g of omega-3 fatty acids daily for 6 weeks (28). They observed improvement in body weight, energy, and macronutrient intake, and nutritional laboratory outcomes (albumin and transferrin) in the active intervention group compared to placebo. The low sample size was the main limitation of the mentioned study that was partially resolved in our study. The low number of patients was mainly due to limiting the enrollment of patients to chemotherapy that was not applied in our study. Our study shows that the beneficial effects of omega-3 fatty acids are not limited to the prevention of chemotherapy-associated side effects.

Besides nutritional status, it is shown that omega-3 can modulate immune and inflammatory responses in patients with cancer. Feijó et al. have evaluated the effect of omega-3 supplementation on immune and inflammatory responses and nutritional status of patients with gastric cancer during the chemotherapy pre-treatment course (29). They have used CRP and IL-6 as main indicators of inflammatory response in these patients that were significantly decreased in patients receiving omega-3 fatty acids compared to control. However, our study failed to show any significant decrease in the level of IL-6 in the patients receiving active supplementation compared to control. This difference between the results of this study and the study by Feijó et al. may be due to the differences in enrolled populations. Patients in the study of Feijó et al. had gastric cancer and had a higher baseline level of IL-6 compared with our patients who had lung cancer with a lower baseline level of IL-6. The enrolled patients in the study of Feijó et al. were receiving chemotherapy pre-treatment courses leading to an increase in the level of IL-6 but that was not the case in our study. Another difference between the two studies was that besides CRP we have measured TNF-α for the evaluation of the anti-inflammatory effect of omega-3 fatty acids. TNF-α is a cytokine with a great role in tumor immune surveillance that has a significant contribution to the progression of cancer (30). It is previously shown that TNF-α is associated with weight reduction, anemia, and poor nutritional status in patients with cancer (31). Our study also showed the suppression of TNF-α in patients with lung cancer receiving omega-3 supplementation, which improved nutritional status.

Besides the mentioned studies, pre-clinical and clinical studies have evaluated the effect of omega-3 fatty acids in lung cancer. An *in vitro* study by Bai et al. showed that DHA can suppress the growth and invasion in non-small cell lung cancer cell line, which was mediated by resolvin D1/microRNA-138-5p/Forkhead Box C1 (RvD1/miR-138-5p/FOXC1) pathway (32). Another report by Yang et al. showed that the tumor growth inhibition of omega-3 is through the downregulation of Protein kinase B (Akt) phosphorylation by Prostaglandin E3 (PGE_3_) (33). In a human study reported by Finocchiaro et al., the effect of omega-3 fatty acid supplementation is evaluated on inflammatory and oxidative stress markers in 33 patients with lung cancer in a 66 days randomized clinical trial. They have reported positive results in suppression of inflammation and oxidative stress in patients receiving omega-3 supplementation compared to placebo (34). Their result on the anti-inflammatory effects of omega-3 was in concordance with our result. Another finding reported by Lu et al. has approved the anti-inflammatory effect of the omega-3 fatty acids in 137 patients with advanced non-small cell lung cancer through suppression of serum CRP and IL-6 level but failed to show any effect on the nutritional status of the patients (35). However, in contrast to these results, our findings showed no significant change in IL-6 but the beneficial effect in some outcomes of nutritional status of patients. The difference may be due to the enrolled patient population, which were patients with advanced non-small cell lung cancer in the study by Lu and the earlier stage of all patients with a different type of lung cancer in our study. Besides the mentioned studies, other trials showed that n-3 fatty acids can improve quality of life, functional status (36), and nutritional status (37) in patients with lung cancer. It is also shown that skeletal muscle depletion is associated with a reduced level of n-3 serum fatty acids in patients with lung cancer (38).

Besides the observed effect of omega-3 supplementation on the nutritional status of patients with different types of cancer, the effect of this nutritional supplement on the prevention or treatment of cancer itself is controversial. An Umbrella Systematic Review and Meta-Analysis published in 2020 statistical methods were used to sum up the results of the studies on the effect of omega-3 on the risk of different types of cancer, such as prostate, liver, brain, skin, endometrial, and breast cancers (39). In this review, more than 57 meta-analyses were included with conflicting results. They finally concluded that current evidence is not enough to support the preventive effect of omega-3 supplementation in the development of cancer. So the results of our study should not be interpreted as the preventive or therapeutic effect of omega-3 fatty acid supplementation in patients with lung cancer. It is only shown that this nutritional supplement can improve the nutritional status of the patients through suppression of inflammatory response that can be considered as palliative care in these patients being able to increase their quality of life.

Besides the strength of using a prospective interventional approach, and randomizing and blinding the enrolled patients in our study, this study suffers some important limitations. Application of only nutritional status and inflammatory markers as outcomes prevented us to make any conclusion about the effect of the intervention on final important outcomes such as mortality rate (40, 41). Consideration of such outcomes needs long-duration follow-up, which was not possible in our protocol. The small number of enrolled patients was another limitation that arises the need of performing further trials with a larger sample size to better evaluate the results. Including only patients with high risk or at risk for nutritional problems based on the Nutrition Risk Screening 2002 tool limits the generalizability of the results to the population of these patients. The difference in baseline gender ratio should also be considered in the interpretation of the results. However, there is no available evidence on the gender difference in omega-3 fatty acids in patients with cancer. The history of smoking is not considered, and the amount of dietary PUFAs is not calculated in the enrolled patients, which are expected to be similar in the two study groups based on the random allocation. It is also important to consider that in this trial a mixed formulation of EPA and DHA was compared with placebo, and we could not compare the effect of every single compound in the observed outcome. Taking into consideration the mentioned limitations, the results of our study showed the beneficial effects of omega-3 fatty acid supplementation on nutritional status, including both anthropometric and laboratory indices, of patients with lung cancer. The study also showed the suppression of systemic inflammatory response evaluated by serum CRP level and TNF-α level that may be associated with the observed effect on the nutritional status of these patients.

## Data Availability Statement

The original contributions presented in the study are included in the article/supplementary material, further inquiries can be directed to the corresponding author.

## Ethics Statement

The studies involving human participants were reviewed and approved by Local Medical Ethics Committee of Anhui Medical University. The patients/participants provided their written informed consent to participate in this study.

## Author Contributions

MC and SZ designed the work. MC, SZ, and CN included and followed the patients. QH analyzed the data, wrote the manuscript, and supervised the work. All authors critically revised and approved the final version of the manuscript.

## Conflict of Interest

The authors declare that the research was conducted in the absence of any commercial or financial relationships that could be construed as a potential conflict of interest.

## Publisher's Note

All claims expressed in this article are solely those of the authors and do not necessarily represent those of their affiliated organizations, or those of the publisher, the editors and the reviewers. Any product that may be evaluated in this article, or claim that may be made by its manufacturer, is not guaranteed or endorsed by the publisher.
